# Inducible Selectable Marker Genes to Improve *Aspergillus fumigatus* Genetic Manipulation

**DOI:** 10.3390/jof7070506

**Published:** 2021-06-24

**Authors:** Clara Baldin, Alexander Kühbacher, Petra Merschak, Luis Enrique Sastré-Velásquez, Beate Abt, Anna-Maria Dietl, Hubertus Haas, Fabio Gsaller

**Affiliations:** Institute of Molecular Biology, Biocenter, Medical University of Innsbruck, 6020 Innsbruck, Austria; clara.baldin@i-med.ac.at (C.B.); alexander.kuehbacher@i-med.ac.at (A.K.); petra.merschak@i-med.ac.at (P.M.); luis.sastre@i-med.ac.at (L.E.S.-V.); Sigrid.Abt@i-med.ac.at (B.A.); anna_maria.dietl@hotmail.com (A.-M.D.)

**Keywords:** dominant selectable marker, *Aspergillus fumigatus*, inducible marker, *hph*, *ptrA*, xylanase promoter, thiamine, hygromycin, pyrithiamine

## Abstract

The hygromycin B phosphotransferase gene from *Escherichia coli* and the pyrithiamine resistance gene from *Aspergillus oryzae* are two dominant selectable marker genes widely used to genetically manipulate several fungal species. Despite the recent development of CRISPR/Cas9 and marker-free systems, in vitro molecular tools to study *Aspergillus* *fumigatus*, which is a saprophytic fungus causing life-threatening diseases in immunocompromised hosts, still rely extensively on the use of dominant selectable markers. The limited number of drug selectable markers is already a critical aspect, but the possibility that their introduction into a microorganism could induce enhanced virulence or undesired effects on metabolic behavior constitutes another problem. In this context, here, we demonstrate that the use of *ptrA* in *A. fumigatus* leads to the secretion of a compound that allows the recovery of thiamine auxotrophy. In this study, we developed a simple modification of the two commonly used dominant markers in which the development of resistance can be controlled by the xylose-inducible promoter *PxylP* from *Penicillium chrysogenum*. This strategy provides an easy solution to avoid undesired side effects, since the marker expression can be readily silenced when not required.

## 1. Introduction

*Aspergillus fumigatus* is a ubiquitous saprophytic fungus that also represents the primary cause of life-threatening invasive aspergillosis in immunocompromised hosts. In addition, *A. fumigatus* is also known to affect immunocompetent individuals, inducing chronic and allergic diseases [1]. The characterization of virulence-related traits and molecular features of human as well as plant pathogens relies heavily on genetic manipulation. The characterization of *A. fumigatus* still extensively depends on the use of dominant selectable markers, particularly the hygromycin B phosphotransferase (*hph*) gene and the pyrithiamine resistance (*ptrA*) gene. *hph*, which confers resistance to hygromycin, encodes a phosphotransferase from *Escherichia coli*, and is one of the most commonly utilized dominant selectable markers to genetically modify organisms, from bacteria to mammalian cells [2,3,4,5,6,7,8,9]. For use in *Aspergilli*, the *hph* gene is usually under control of the *Aspergillus nidulans gpdA* promoter [10]. *ptrA* was characterized in *Aspergillus oryzae* after screening for pyrithiamine-resistant strains [11]. Pyrithiamine resistance is acquired by a point mutation in the 5′ UTR region of *thiA*, which is a thiamine thiazole synthase encoding gene related to *Saccharomyces cerevisiae THI4*. The *thiA* upstream region has been identified as a riboswitch in thiamine synthesis regulation, but the underlying mechanism by which resistance is conferred has still not yet been fully characterized [12,13]. However, this has not prevented the (extensive) use of *ptrA* as selectable markers in different *Aspergillus* as well as *Trichoderma* and *Penicillium* species [14,15,16,17].

A major drawback of drug resistance markers nowadays is related to the generation of genetically modified organisms, but this problem mostly concerns species that are industrially relevant [18,19,20]. In the case of *A. fumigatus*, which is not used in production processes and is studied for its effect on human health as pathogen, drug resistance markers have the advantages of not needing a specific host strain, allowing the stable integration of constructs with high efficiency and generating mutant strains that are easy to select and purify [7,16].

Another important aspect to consider is that the constant expression of exogenous constructs inserted in the microbial genome can affect the overall metabolism of the studied species. Normally, this problem can be circumvented by the appropriate use of control strains, but it is rare in the case of virulence studies to include a strain carrying the dominant marker as a control. A significant example was described by Smulian et al. [8], when the *hph* was proved to affect virulence in *Histoplasma capsulatum*, despite the successful employment of such a marker in previous studies. Similar reports are rare, especially due to the fact that virulence studies normally involve the use of animal models for which implementation of the three Rs rule (Replacement, Reduction, Refinement) is essential.

Recently, we became aware that the *ptrA* marker could also affect the secretome of *A. fumigatus*. In this work, we were able to prove that *A. fumigatus* strains genetically modified with the help of the *ptrA* marker cassette were unexpectedly secreting metabolites in the medium, affecting the growth of other strains plated in their proximity. In light of this discovery, we decided to upgrade the commonly used dominant markers *hph* and *ptrA* to their respective inducible versions by expressing these resistance genes under the control of the xylanase promoter *PxylP* derived from *Penicillium chrysogenum* [21]. This modification of the dominant marker systems permits investigators to switch on gene expression only when required, for example during the transformation or the selection process, leaving otherwise the marker cassette silenced. Notably, *PxylP* has been shown to be inactive during standard murine infection conditions unless xylose is supplied in the drinking water; i.e., it enables in vivo (fine) tuning of *A. fumigatus* gene activity [22].

## 2. Materials and Methods

### 2.1. Strains, Media and Growth Conditions

The strains used in this study are listed in Table 1. *A. fumigatus* A1160P+ [23] was used as the parental strain and referred to as wild-type (wt), while *A. nidulans* A52 was used as reporter strain; since it solely served the purpose of a reporter, the use of a different background species was considered acceptable.

Spores were amplified on Sabouraud dextrose (SAB, Sigma-Aldrich Corp., St. Louis, MI, USA) medium for 3 days at 37 °C. *Aspergillus* minimal medium (AMM) [24] containing 1% glucose as carbon source and 20 mM ammonium tartrate as nitrogen source was used as standard medium in the assays. To create *PxylP*-inducing conditions, xylose was provided. For experiments involving A52, biotin was supplemented at a final concentration of 0.25 μg/mL.

**Table 1:** Fungal strains used in this study.

Strains	Genotype	Reference
A1160P+(wt)	∆*ku80, pyrG+*	Fraczek et al. [23]
A52	FGSC A52 (bi1 thi4)	ATCC^®^ 24761™
Δ*fcyB-hph*	Δ*fcyB::hph*	Gsaller et al. [25]
Δ*fcyB-ptrA*	Δ*fcyB::ptrA*	This study
Δ*fcyB-hph*^xyl^	Δ*fcyB::PxylP-hph*	This study
Δ*fcyB-ptrA*^xyl^	Δ*fcyB::PxylP-ptrA*	This study
Δ*pksP-hph*^xyl^	Δ*pksP::PxylP-hph*	This study
Δ*pksP-ptrA*^xyl^	Δ*pksP::PxylP-ptrA*	This study

### 2.2. Generation of Transformation Constructs

Oligonucleotides used for the generation of transformation constructs are listed in Table S1 (Supplementary Materials). In order to delete *fcyB* by the insertion of *ptrA*, we followed the strategy previously used to delete the same gene by inserting the *hph* marker cassette [25]. Briefly, the marker sequence was amplified via PCR using primers ptrA 5 and ptrA 3 using the plasmid pSK275 as template. The flanking regions upstream and downstream of the *fcyB* coding sequence required for homologous recombination were amplified from wt genomic DNA using primers fcyB-1 and fcyB-2RV for the 5′ flanking region and primers fcyB-3 and fcyB-4RV for the 3′ flanking region. The three fragments thus generated were linked via fusion PCR using the nested primers fcyB-N1 and fcyB-N2 (Figure S1a).

For the deletion of *fcyB* by insertion of the inducible marker cassettes, instead, a plasmid was first generated as starting point for further modifications, using the strategy previously described by Birštonas et al. [26]. Briefly, primers BB-pfcyB-FW and BB-pfcyB-RV were used to amplify the backbone from the template plasmid pfcyB [26], while the cassette containing *mKate2*^PER^ coding sequence under the control of the inducible promoter *PxylP* was PCR amplified with primers pX-cass-FW and pX-cass-RV using pX-mKate2^PER^ [26] as a template. Then, the inducible cassette was cloned into the backbone using overlapping regions introduced in the PCR step. The resulting plasmid, named pΔfcyB_mKate2^xyl^, was used as a template for the generation of the hygromycin and pyrithiamine inducible constructs. Primers pX-FW.2 and pX-RV.2 were used to amplify the backbone from pΔfcyB_mKate2^xyl^. The coding sequence of the marker genes was generated using as template pAN 7-1 with the primers hphxyl-FW and hphxyl-RV, for the hygromycin, and pSK275 with the primers ptrA_FW and ptrA_RV, for the pyrithiamine construct. Then, the PCR products were subcloned into the backbone with the help of the overlapping regions introduced during the PCR step, generating pΔfcyB_hph^xyl^ and pΔfcyB_ptrA^xyl^. These two plasmids were linearized via restriction digestion with *Not*I for homologous recombination at the *A. fumigatus fcyB* locus (Figure S1b).

To delete *pksP* via the insertion of either the inducible hygromycin or inducible pyrithiamine cassette, the marker was PCR amplified using the primers fcyB-GFPxyl-FW and fcyB-GFPxyl-RV from pΔfcyB_hph^xyl^ and pΔfcyB_ptrA^xyl^, respectively. The flanking regions upstream and downstream of *pksP* required for homologous recombination were amplified from *A. fumigatus* wt genomic DNA using the primers 5′pksP-FW and 5′pksP-RV for the 5′ flanking region and the primers 3′pksP-FW and 3′pksP-RV for the 3′ flanking region. The inducible marker cassette of choice was linked to the flanking regions via fusion PCR, using nested primers pksP-N1 and pksP-N2 (Figure S1c).

### 2.3. Fungal Transformation

Fungal transformations were done according to the protocol previously described [23] with slight modifications. For all transformations, 1 μg of the respective DNA constructs was used. Plasmids were linearized via restriction digestion with *Not*I, while fragments resulting from fusion PCR were ready to use. Both PCR and digestion products were purified with the Monarch kit (New England Biolabs Inc., Ipswich, MA, USA) according to the manufacturer’s instructions and checked on a 1% agarose gel. Deletion mutants for *fcyB* were selected using 10 μg/mL of flucytosine on AMM plus 1 M sucrose and 0.1 M citrate buffer pH5 to maintain acidic pH [26]. Transformation using either pyrithiamine (0.1 μg/mL) or hygromycin (200 μg/mL) was carried out on AMM plus 1 M sucrose. For transformation of the inducible marker constructs, glucose was omitted, and 1% xylose was added to the medium.

### 2.4. Nucleic Acid Manipulation, Southern and Northern Blot Analysis

Circular plasmids from *E. coli* were extracted using the Monarch Plasmid Miniprep Kit (New England Biolabs Inc.).

For PCR amplification, Q5 High Fidelity DNA Polymerase (New England Biolabs Inc.) was employed according to the manufacturer’s instructions.

*A. fumigatus* transformants were verified via Southern blot analysis. About 500 ng of genomic DNA was incubated for restriction digestion overnight, and the different bands were separated on a 1% agarose gel.

For Northern blot analysis, *A. fumigatus* strains were grown in 100 mL liquid cultures for 16 h at 37 °C. The medium used contained 1% sucrose as the carbon source. For *PxylP*-driven induction, 1% xylose was added. RNA extraction was performed with TRI Reagent (Sigma-Aldrich Corp., St. Louis, MI, USA) according to the manufacturer’s instructions, and 10 μg of RNA was used for electrophoresis on a 0.6 M formaldehyde agarose gel. Northern blot analysis was carried out as described previously, using digoxigenin-labeled probes [27].

### 2.5. Spot Assays on Plate

To assess radial growth, 10^4^ spores in a total volume of 5 μL were spotted onto AMM [24] agar in the absence and presence of the selecting agent. Hygromycin was tested at two different concentrations of 100 μg/mL and 200 μg/mL, based upon relevant data from different laboratories, while pyrithiamine was used at a fixed concentration of 0.1 μg/mL. Either glucose or sucrose was used as carbon source, both at a concentration of 1%. Xylose was supplemented as an inducer at concentrations of 0.1% and 1%. Then, plates were incubated at 37 °C for 48–72 h.

### 2.6. Microtiter-Plate Assays

Supernatant from different strains was tested in order to verify the presence of secreted metabolites able to recover A52 thiamine auxotrophy. Therefore, 100 mL of liquid AMM containing 1% sucrose as carbon source and, in the case of inducing conditions, additionally 1% xylose, was inoculated with the desired spores in order to obtain a final concentration of 10^6^ spores/mL. The cultures were incubated overnight at 37 °C shaking at 200 rpm. Aliquots from the supernatant were collected after 18 h and preserved at −80 °C upon usage. Nunc96 plates (Thermo Scientific Inc., Waltham, MA, USA) were used for testing the growth of the wt and the reporter strain A52. Spores were inoculated in 2X AMM to obtain a final concentration of 2 × 10^5^ spores/mL. Each well contained 50 μL of the media/spore solution and 50 μL of the supernatant to test (or of thiamine at different concentrations for the titration experiments). Plates were incubated at 37 °C for 20 h and then scanned with the IncuCyte S3 Live-Cell Analysis System equipped with a 20× magnification S3/SX1 G/R Optical Module (Essen Bioscience Inc., Ann Arbor, MI, USA). Fungal growth was analyzed using the Basic Analyzer tool of the IncuCyte S3 software (Version 2020; Essen Bioscience Inc.) for confluence % (Segmentation adjustment: 0; Adjust Size: 0). A confluence mask was exported with each respective image for a better visualization of cells in contrast to the background.

## 3. Results and Discussion

### 3.1. The Pyrithiamine Resistance Cassette Induces the Production of One or More Metabolites Able to Complement Thiamine Auxotrophy

**Figure 1:**
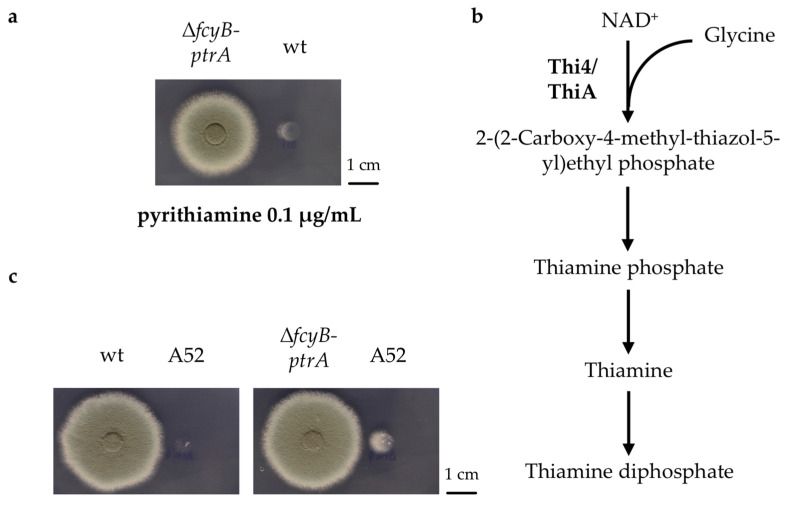
Proximity effect of *A. fumigatus* strain carrying the *ptrA* cassette. (**a**) Spot assay on solid AMM containing 0.1 μg/mL pyrithiamine showing partial growth of the wt strain in proximity to Δ*fcyB-ptrA*. (**b**) Simplified version of the thiamine biosynthetic pathway, adapted from Dietl et al. [28]. (**c**) Spot assay on solid AMM using A52 as reporter strain spotted in proximity to either wt or Δ*fcyB-ptrA*.

During the course of previous experiments, we observed that the *A. fumigatus* wt strain was able to partially grow in the presence of 0.1 μg/mL pyrithiamine if it was spotted in the proximity of mutant strains carrying the *ptrA* marker cassette. We hypothesized that *ptrA* introduction into *A. fumigatus* modifies the fungal metabolism and induces the secretion of an unidentified metabolite able to confer partial pyrithiamine resistance to an adjacent pyrithiamine-sensitive strain. In order to prove this hypothesis, a *ptrA* construct was inserted into the previously described endogenous marker locus *fcyB*, generating Δ*fcyB-ptrA* [26]. As is apparent in Figure 1a, the wt strain spotted in close proximity to Δ*fcyB-ptrA* showed slight growth on AMM supplemented with pyrithiamine, particularly on the side closest to Δ*fcyB-ptrA*.

Considering that *ptrA*-mediated resistance to pyrithiamine is related to the regulation of thiamine metabolism, we decided to investigate mutant strains with defects in key enzymes related to thiamine biosynthesis (Figure 1b) such as *A. nidulans* A52 (ATCC^®^ 24761™), which is lacking the thiamine thiazole synthase encoding gene *thi4* (AN3928). When spotted on AMM in proximity to the wt strain, A52 was not able to grow, but when in proximity of Δ*fcyB-ptrA,* A52 showed a partial recovery of its growth phenotype particularly on the side closer to the *ptrA*-carrying strain, generating a crescent moon shape (Figure 1c).

Taken together, these results suggested that expression of the *ptrA* marker cassette in *A. fumigatus*, in our example in Δ*fcyB-ptrA*, causes the release of a metabolite that promotes the growth of wt strains in the presence of pyrithiamine and allows the recovery of the thiamine auxotrophic A52 strain.

**Figure 2:**
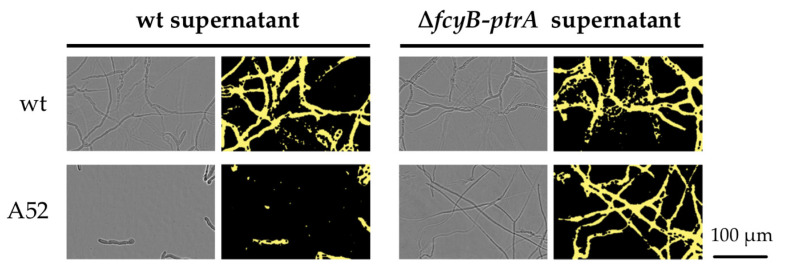
Representative images showing the growth promotion of the reporter strain A52 using *A. fumigatus* wt (**top**) and Δ*fcyB-ptrA* (**bottom**) culture supernatants collected after 18 h growth. wt was used as a normal growth reference. Phase images (**left**) are shown for better visualization together with the respective confluence mask images (**right**).

In order to confirm this finding, a feeding assay involving supernatant from *A. fumigatus* cultures has been set up, using A52 as a reporter strain and wt as positive control. Samples of supernatant from wt and Δ*fcyB-ptrA* liquid cultures were collected after 18 h of growth at 37 °C and tested for its ability to promote growth in the tested strains. As shown in Figure 2, the supernatant of wt culture was not permissive of A52 growth, while Δ*fcyB-ptrA* supernatant enables A52 to fully germinate and produce well-branched hyphae.

These data further validate that the introduction of the marker cassette *ptrA* in *A. fumigatus* leads to the secretion of a thiamine pathway metabolite able to recover thiamine auxotrophy caused by a defect in the *thi4* gene.

### 3.2. Generation of A. fumigatus Strains with Inducible Resistance Marker Cassettes

To overcome the unwanted side effects caused by the overexpression of a selectable marker gene, xylose-inducible versions of both *hph* and *ptrA* were generated by expressing these resistance genes under control of the promoter *PxylP* with the aim of switching on the expression of the marker gene on demand. For consistency, we decided to integrate the inducible alleles, termed *hph*^xyl^ and *ptrA*^xyl^, via homologous recombination at the *fcyB* locus, using flucytosine selection for the transformation. The generated strains, Δ*fcyB-hph*^xyl^ and Δ*fcyB-ptrA*^xyl^, were screened via Southern blot (Figure S2).

**Figure 3:**
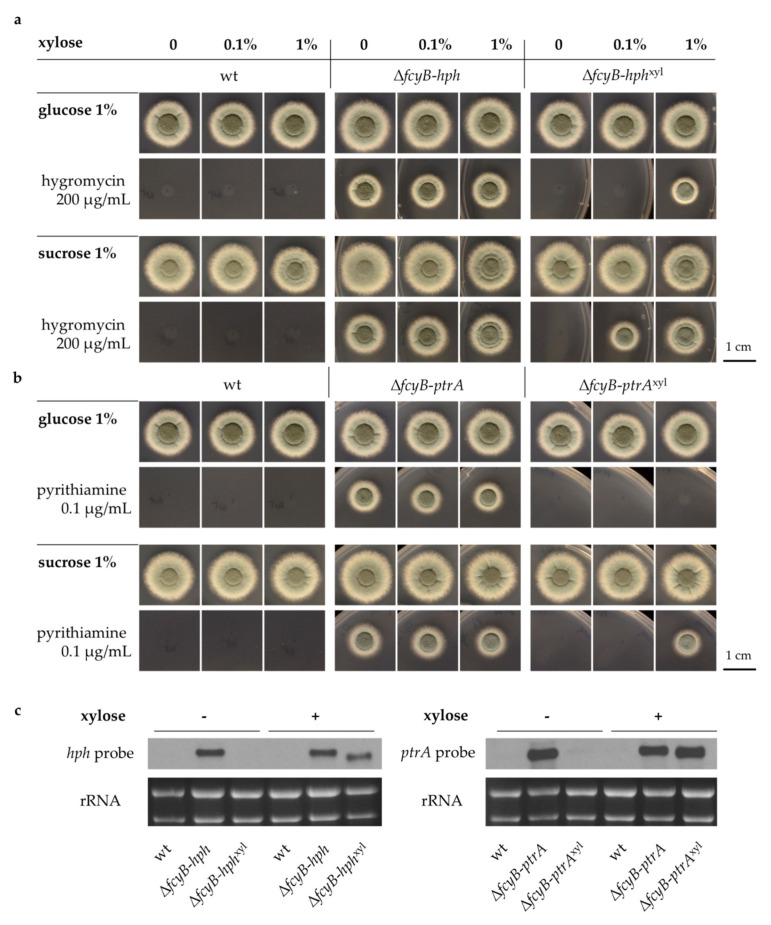
Characterization of inducible marker strains in comparison to their respective non-inducible version in the presence of different xylose concentrations. (**a**) Representative images of a hygromycin susceptibility growth assay for wt, Δ*fcyB-hph*, and Δ*fcyB-hph*^xyl^ in the presence of xylose concentrations of 0%, 0.1%, and 1%, with either glucose or sucrose as a carbon source. (**b**) Representative images of a pyrithiamine susceptibility growth assay for wt, Δ*fcyB-ptrA*, and Δ*fcyB-ptrA*^xyl^ in the presence of xylose concentration of 0%, 0.1%, and 1%, with either glucose or sucrose as a carbon source. (**c**) Northern blot analysis showing a comparison of the transcript levels of *hph* (**left**) and *ptrA* (**right**) in wt and drug-resistant strains in their non-inducible or inducible versions upon induction with 1% xylose and sucrose as a carbon source.

Plate growth-based susceptibility assays were initially performed to verify the ideal conditions for further experiments with the inducible strains (Figure 3a,b). Considering that *PxylP* is repressed by glucose [21], the common carbon source used in AMM, we decided to also test a different medium containing 1% sucrose, which is used as an osmotic stabilizer for protoplasts during transformation, instead of glucose. Δ*fcyB-hph*^xyl^ was compared to wt and Δ*fcyB-hph* on AMM plates containing increasing concentrations of xylose in the absence and presence of hygromycin (Figure 3a). Employing our background strain, we verified that 100 μg/mL of the drug was not sufficient to completely repress the growth of the wt or the inducible strain Δ*fcyB-hph*^xyl^ under non-inducing conditions (Figure S3). The wt strain was unable to grow with the presence of 200 μg/mL hygromycin in the medium, whereas Δ*fcyB-hph* grew well in all conditions, and Δ*fcyB-hph*^xyl^ displayed growth in a manner dependent on both the xylose concentration and the major carbon source used. When glucose was used, a minimum concentration of 1% xylose was necessary to permit Δ*fcyB-hph*^xyl^ sporulation and growth, while in the presence of sucrose, 0.1% xylose was sufficient. The same experiment was performed with the pyrithiamine-resistant strains, both inducible and non-inducible, and it revealed even more interesting results (Figure 3b). The wt strain was not able to grow in the presence of pyrithiamine at the standard concentration of 0.1 μg/mL while Δ*fcyB-ptrA* was resistant to the drug. The inducible strain Δ*fcyB-ptrA*^xyl^ showed susceptibility to pyrithiamine even at a xylose concentration of 1% when glucose was used as a carbon source, indicating the need for high levels of gene expression in order for drug resistance to manifest. Instead, when sucrose was used, 1% xylose was sufficient to promote Δ*fcyB-ptrA*^xyl^ growth. Based on these results, all further experiments involving the inducible marker strains were conducted using sucrose exclusively as the carbon source in the media and a xylose concentration of 1% for inducing conditions.

Our test indicated that to induce the desired resistance phenotype when using inducible marker cassettes under the regulation of *PxylP*, different concentrations of xylose might be required. It is particularly relevant to note that the regulatory mechanism of *PxylP* is known to be repressed by glucose and induced by xylose. In general, for genes that do not require a high expression level, the combination of xylose with glucose in the medium might also be beneficial [21,29], but in the case of resistance genes such as *ptrA*, the expression level is particularly relevant, since effective neutralization of the selective drug is contingent on the presence of sufficient quantities on gene product. We could verify that to allow growth in the presence of 200 μg/mL hygromycin, a minimum concentration of 1% xylose was necessary when glucose was present as the carbon source, whilst 0.1% xylose was sufficient when using sucrose. However, in the presence of 0.1% µg/mL pyrithiamine, higher levels of marker transcript were necessary, as revealed by the observation that in order to promote growth to a comparable level to that seen for the standard *ptrA* cassette (Δ*fcyB-ptrA*), a complete absence of glucose and induction with 1% xylose was required.

Northern blot analysis confirmed the upregulation of both *hph* and *ptrA* transcript levels during inducing conditions when using *PxylP* and showed the absence of transcript in the inducible strain when xylose was not provided (Figure 3c). Previous studies have identified a potential ‘leakiness’ of *PxylP* [21,22], whereby the regulated expression of the target genes was not completely achieved due to basal low-level expression. However, in our experiments, this potential low-level expression appeared to be negligible.

**Figure 4:**
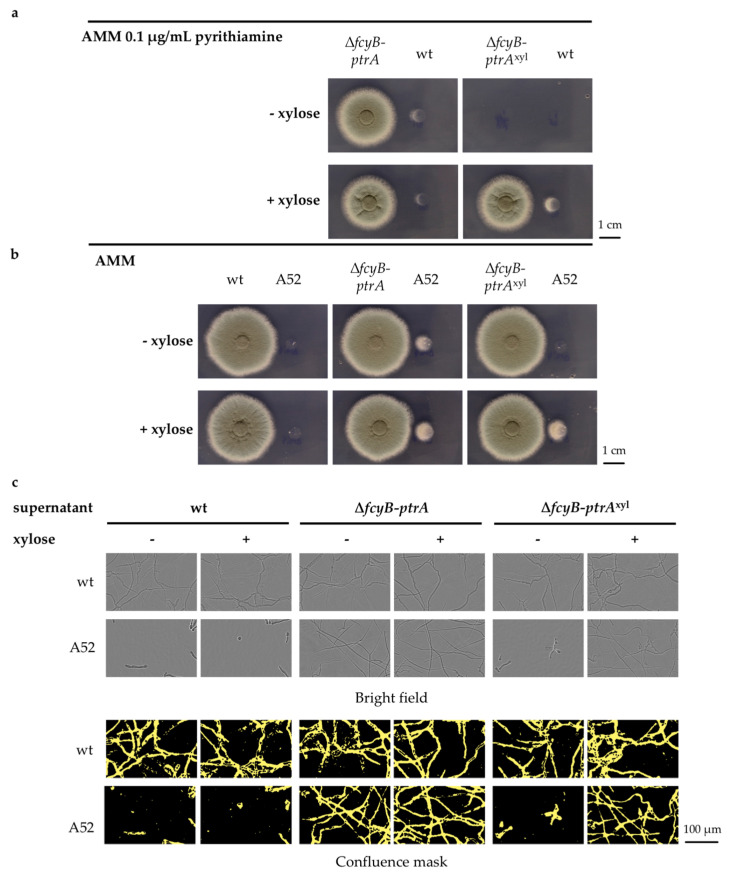
Effect of *ptrA* cassette, both inducible and non-inducible, on fungal growth. (**a**) Spot assay on solid AMM containing 0.1 μg/mL pyrithiamine showing the proximity effect of the *ptrA-*carrying strain Δ*fcyB-ptrA* (**left**) or Δ*fcyB-ptrA*^xyl^ (**right**) on the wt both in the absence and presence of xylose. (**b**) Spot assay on solid AMM using A52 as a reporter strain in proximity to wt, Δ*fcyB-ptrA*, or Δ*fcyB-ptrA*^xyl^ in the absence and presence of xylose. (**c**) Representative images showing fungal growth of A52 growth after 20 h in liquid AMM at 37 °C using *A. fumigatus* wt, Δ*fcyB-ptrA*, and Δ*fcyB-ptrA*^xyl^ culture supernatants collected at 18 h in either the presence or absence of xylose. Phase images (**top**) are shown for better visualization together with the respective confluence mask images (**bottom**).

Δ*fcyB-ptrA*^xyl^ was subsequently compared to Δ*fcyB-ptrA* concerning its capacity to secrete the unidentified metabolite that could confer resistance to pyrithiamine in wt and recover *thi4* auxotrophy in A52. When spotted on AMM containing 0.1 μg/mL pyrithiamine, the wt in proximity to Δ*fcyB-ptrA* was able to partially grow both in the presence and absence of xylose. Δ*fcyB-ptrA*^xyl^ itself was unable to grow in the presence of pyrithiamine if xylose was omitted, but upon induction, it was able to grow and to allow partial growth of the wt (Figure 4a). When using A52 as a reporter on AMM without selection, the wt was not able to recover the *thi4* auxotrophy independent of the specific growth conditions. As expected, Δ*fcyB-ptrA* enabled A52 to partially grow both in the presence and absence of xylose. Consistent with our expectations of *ptrA* overexpression during inducing conditions, Δ*fcyB-ptrA*^xyl^ was only able to induce the growth of A52 when xylose was present in the medium (Figure 4b). This result further suggests that *ptrA* overexpression mediated by the original *ptrA* marker cassette leads to an enhanced secretion of metabolites, thereby rescuing *thi4* auxotrophy independently of the growth medium used, whereas the enhanced production and/or extracellular secretion of the unidentified metabolite in strains carrying the inducible variant is restricted to inducing conditions. This experiment also confirmed that secretion of the thiamine auxotrophy-salvaging metabolite is independent of *fcyB* deletion but strictly reliant on *ptrA* expression, since under non-inducing conditions, Δ*fcyB-ptrA*^xyl^ could not promote A52 growth.

Following the set of experiments previously performed for Δ*fcyB-ptrA*, a further analysis was conducted using 96-well plates in order to test for the secretion of the thiamine equivalent in liquid culture. A52 was used as reporter strain and supernatants from wt, Δ*fcyB-ptrA*, and Δ*fcyB-ptrA*^xyl^ liquid cultures, which were grown in the presence and absence of xylose with 1% sucrose as a carbon source, were collected after 18 h. In agreement with previous results observed using solid AMM, only the supernatant from Δ*fcyB-ptrA* in the presence and absence of xylose and Δ*fcyB-ptrA*^xyl^ with xylose contained sufficient amounts of the secreted metabolite(s) to enable A52 growth (Figure 4c).

**Figure 5:**
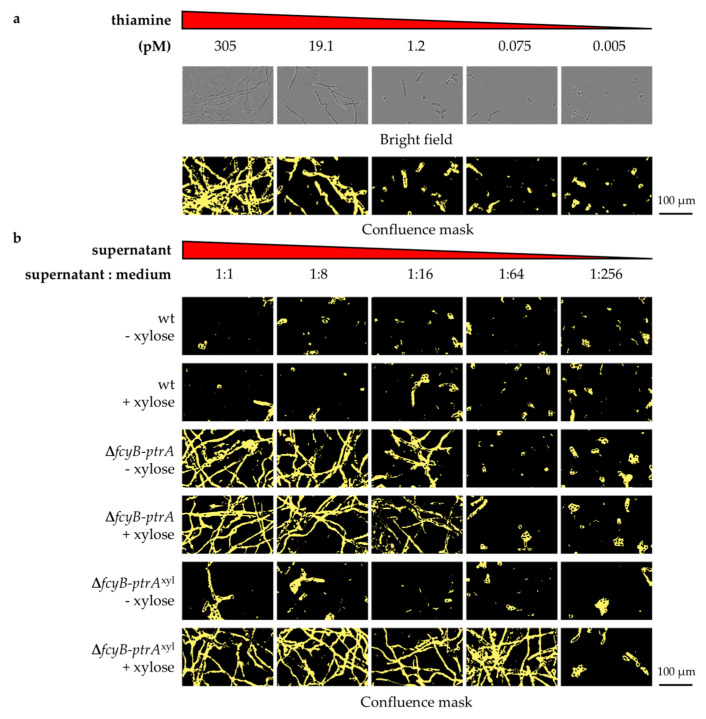
Fungal growth of reporter strains according to thiamine or putative thiamine equivalent concentration in the medium. Thiamine or culture supernatant were subjected to a serial dilution procedure starting with 1:1 dilution of supernatant with medium in well number 1. (**a**) A52 growth in the presence of decreasing concentrations of thiamine. The complete dilution series is shown in Figure S4. (**b**) A52 growth pattern in the presence of decreasing concentrations of *A. fumigatus* wt, Δ*fcyB-ptrA,* and Δ*fcyB-ptrA*^xyl^ supernatants from 18 h cultures either in the presence or absence of xylose. For simplicity, phase images are omitted, and only confluence mask images are shown. Phase images and complete dilution series are shown in Figure S5.

In order to obtain a general estimate for the thiamine equivalent accumulation in culture media, we monitored the A52 strain growth pattern observed when using serial dilutions of wt, ∆*fcyB-ptrA,* and ∆*fcyB-ptrA*^xyl^ supernatants and compared its growth recovery to that detected when employing a thiamine titration assay. After 20 h incubation at 37 °C, the minimum thiamine concentration required to promote A52 growth was 9.54 pM (Figure 5a). This was comparable to a dilution of culture supernatant in fresh medium of 1:8 for ∆*fcyB-ptrA* without xylose, 1:16 for ∆*fcyB-ptrA* with xylose, and 1:64 for ∆*fcyB-ptrA*^xyl^ in the presence of xylose (Figure 5b). Culture supernatants of wt or ∆*fcyB-ptrA*^xyl^ without xylose did not promote robust fungal growth. A direct comparison of growth promotion by culture supernatants and thiamine is not possible, since it cannot be excluded that secreted thiamine precursors or its phosphorylated derivatives could cure the auxotrophy of A52. Nevertheless, these analyses revealed that the concentration of the unidentified metabolite in culture media is comparable to about 76.3 pM and 152.6 pM thiamine for ∆*fcyB-ptrA* grown in the absence and presence of xylose, respectively, and 610.6 pM thiamine for the xylose-induced ∆*fcyB-ptrA*^xyl^.

Taken together, these findings suggest that the activity of both *hph* and *ptrA* and their conferred resistance can be conditionally induced using *PxylP*.

### 3.3. Validation of the Inducible Marker Cassettes as Selectable Marker in A. fumigatus Transformation

**Figure 6:**
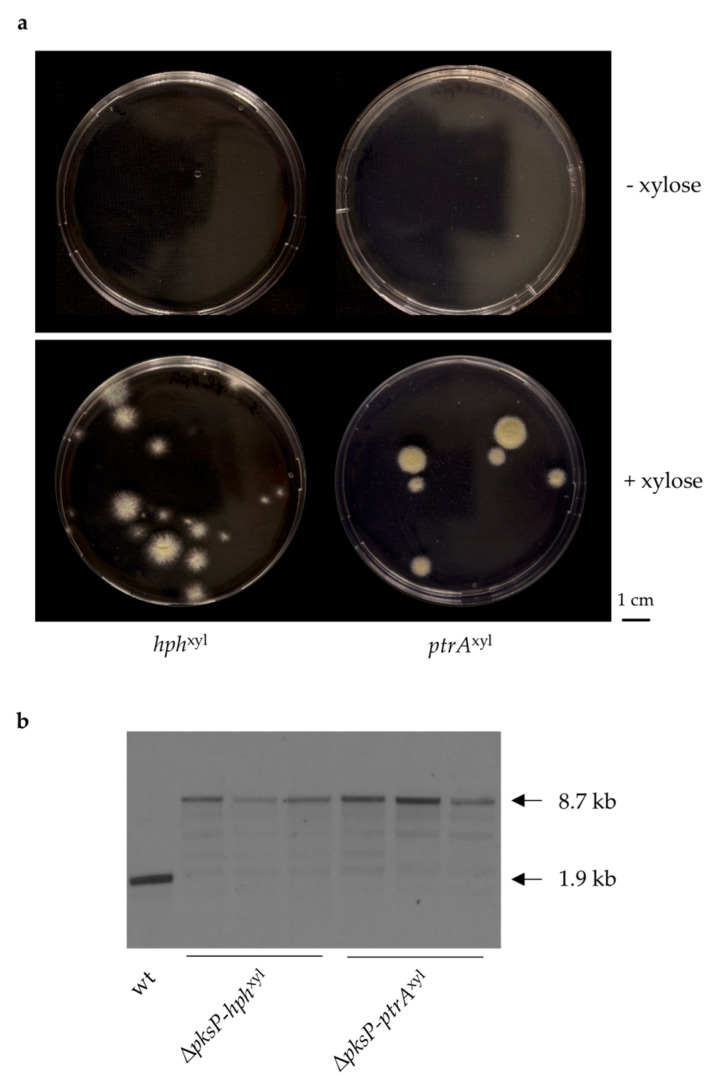
Transformation using *hph*^xyl^ or *ptrA*^xyl^ as a selectable marker. (**a**) Representative transformation plates showing the transformation outcome when using the inducible marker *hph*^xyl^ and *ptrA*^xyl^ using hygromycin or pyrithiamine respectively, for selection. (**b**) Southern blot analysis of three random transformants for each marker in comparison to the wt. Genomic DNA was digested with *BamH*I, and the probe for detection was designed on the *pksP* 3′ flanking region. Expected sizes are reported in the figure.

To confirm the *hph*^xyl^ and *ptrA*^xyl^ cassettes as effective inducible dominant selectable markers in *A. fumigatus*, we employed the marker cassettes to delete the *pksP* gene via homologous recombination in the wt strain. We chose *pksP* as the target to simplify the evaluation of results, since the deletion of this gene generates white colonies [30,31]. In the presence of 1% xylose as an inducer, the inducible *hph* and *ptrA* cassettes generated 15 (14 positive and one negative) and six (all positive) colonies per μg DNA, respectively (Figure 6a). In absence of xylose, no transformant was generated. Three transformants per each construct were randomly selected and confirmed via Southern blot analysis (Figure 6b). The transformation for both constructs has been repeated two further times, providing similar outcomes. All the transformants showed resistance to the respective drug depending on the presence or absence of xylose in the medium.

These results indicate that *hph*^xyl^ and *ptrA*^xyl^ are suitable to be used as dominant markers in *A. fumigatus*, with the advantage compared to the commonly used *hph* and *ptrA* cassettes that the marker gene expression can be induced on demand, e.g., for transformation selection and strain purification, but silenced when no selection is needed. In this way, unwanted cellular effects caused by the selectable marker gene can be avoided simply by its downregulation through the omission of xylose.

## 4. Conclusions

Despite the recent development of marker-free technologies and marker-recycling strategies [32,33,34], dominant selectable markers are still widely used in molecular genetics, particularly for in vitro manipulation [7,35]. Whilst the absence of marker cassettes, in the form of exogenous DNA, presents considerable advantages [36], the possibility to verify the purity of the mutant strains using selective conditions at any time and the transformation efficiency reached when employing dominant markers represent two significant benefits of opting for the transformation approach. Moreover, the characterization of a new gene typically not only requires the generation of a deletion mutant strain but also validation of the gene function through the generation of the corresponding complemented strain that is anticipated to show wt-like behavior. Even considering the use of ∆*ku70/80* derivatives, which already significantly enhanced the homologous recombination events in *A. fumigatus* [37], transformation strategies that are simple, efficient, and do not require the screening of several colonies to identify positive clones are preferable. Typically, these employ the use of selectable marker genes. This often results in the generation of reconstituted strains containing two markers: one used for the deletion and one used for the complementation.

On the other hand, examples highlighting the drawbacks of selectable markers have already been reported, e.g., the locus-dependent effects of the auxotrophic marker *URA3* on hyphal growth and virulence-associated behavior in *Candida albicans* [38]. Even more concerns were raised when the *hph* cassette was proven to affect *H. capsulatum* virulence [8]. Both examples refer to selectable markers that have been successfully used on different occasions, until their undesired effects have materialized.

In this work, we present another example of an unexpected unwanted outcome when introducing a selectable marker: we observed that *A. fumigatus* strains carrying the *ptrA* selectable marker cassette allowed fungal growth on selective media of pyrithiamine-sensitive strains spotted in close proximity. We were able to determine that the *ptrA* marker induced fungal secretion of a metabolite into the medium that was able to recover thiamine auxotrophy induced by *thi4* impairment. Our results suggest that this metabolite could be the product of the Thi4 catalyzed reaction or a metabolite further downstream the thiamine biosynthetic pathway. The elucidation of the specific compound curing the auxotrophy phenotype of the *thi4* defective strain A52 would require further biochemical analysis.

To overcome potential side effect(s) caused by the use of dominant markers without dismissing their undeniable advantages, we decided to upgrade the two most frequently used markers in *A. fumigatus*, the *hph* and *ptrA* cassettes, setting the expression of the resistance gene under the control of the xylanase inducible promoter *PxylP* derived from *P. chrysogenum* [21]. In this way, the presence of the marker cassette should result in negligible expression levels under non-inducing conditions, while the expression of the gene could be switched on by adding xylose as an inducer when required (e.g., for transformation or selection). The two inducible constructs, *hph*^xyl^ and *ptrA*^xyl^, have been first inserted into the *fcyB* locus [26] in order to initially determine the optimal conditions for induction and to obtain similar resistance to the one achieved with the standard *hph* and *ptrA* cassettes. Subsequently, the two inducible constructs have been successfully employed in transformation procedures used to generate *A. fumigatus pksP* deletion mutants, with transformation efficiencies comparable to the ones achieved with the respective non-inducible resistance cassette.

In summary, here, we have demonstrated for the first time that following genomic integration, the expression of the marker gene *ptrA* led to metabolic side effects in *A. fumigatus*. In order to circumvent these undesired effects whilst simultaneously exploiting the benefits of marker system, we developed simple and effective alternatives to the conventional dominant selectable markers, whereby we could avoid unnecessary expression of the marker gene and induce expression on demand and when required—for example, during transformation and purification procedures. We envisage that these systems will abrogate many of the adverse effects associated with the classical dominant marker approach and therefore represent a valuable addition to the fungal researchers’ toolkit.

## Supplementary Materials

The following are available online at https://www.mdpi.com/article/10.3390/jof7070506/s1, Figure S1: Schematic representation of a cloning strategy used to generate transformation constructs, Figure S2: Southern blot analysis of *A. fumigatus* transformants, Figure S3: Hygromycin susceptibility growth assay, Figure S4: Overview of A52 growth over the complete series of thiamine dilutions, Figure S5: Overview of A52 growth over the complete series of serial dilution of supernatants collected after 18 h cultures, Table S1: Primers used in this work.
